# Development of a Chitosan/Cellulose Nanocrystal-Stabilized Pickering Emulsion Film Containing Pomelo Peel Essential Oil and Cinnamaldehyde for Strawberry Preservation

**DOI:** 10.3390/foods15203630

**Published:** 2026-10-13

**Authors:** Juan Li, Qiao-Ling Chen, Yong-Hua Hu, Zhao Long, Xiang-Jin Fu

**Affiliations:** 1 Faculty of Food and Health, Zhangzhou City Vocational College, Zhangzhou 363000, China; 2 National Engineering Research Center for Rice and Byproduct Deep Processing, School of Food Science and Engineering, Central South University of Forestry and Technology, Changsha 410004, China; 3 School of Biological Science and Technology, Minnan Normal University, Zhangzhou 363000, China

**Keywords:** pomelo peel essential oil, Pickering emulsion, active film, biodegradability, strawberry preservation

## Abstract

To develop eco-friendly active packaging for food spoilage control, a Pickering emulsion (PPOEs) was prepared by co-encapsulating pomelo peel essential oil (PPO) and cinnamaldehyde (CIN) (1:1, *v*/*v*) using chitosan (CS) and carboxylated cellulose nanocrystals (C-CNCs) as composite stabilizers. Among the oil-to-water ratios tested, the emulsion prepared at 1:50 showed good storage stability and was selected for film fabrication. This combination exhibited strong antibacterial activity against four common foodborne pathogenic bacteria *(E. coli*, *S. aureus*, *L. monocytogenes*, and *V. parahaemolyticus*) and improved sensory acceptability by effectively masking the pungent odor of CIN with the fresh citrus aroma of PPO. The CS composite film containing PPOEs (CS-30%PPOEs) showed enhanced mechanical properties, UV resistance, and reduced water vapor permeability compared to the pure CS film. During strawberry preservation at 27 ± 4 °C for 5 days, the film (CS-30%PPOEs) resulted in 0% decay, whereas a commercial polyvinyl chloride film (PVC; CK group) resulted in 100% decay. In addition, the composite film reduced weight loss and delayed fruit softening, thereby maintaining post-harvest quality. Soil burial tests over 21 days confirmed good biodegradability. Overall, this study provides a feasible strategy for utilizing pomelo peel waste while achieving a balance of antimicrobial efficacy, sensory quality, and environmental friendliness, offering a promising active packaging solution for controlling post-harvest spoilage of perishable fruits.

## 1. Introduction

Microbial contamination and rapid quality deterioration of perishable fruits during post-harvest storage remain major challenges for the food industry, leading to significant food waste and potential food safety risks [1,2]. Although petroleum-based plastic packaging has been widely used owing to its low cost and good mechanical properties, its non-biodegradability causes severe environmental pollution. Moreover, it does not provide active control over microbial growth on food surfaces [3,4]. Consequently, it is necessary to develop biodegradable active packaging materials that can effectively inhibit spoilage microorganisms and extend the shelf life of fresh produce [5,6].

Chitosan (CS), a cationic polysaccharide derived from crustacean shells, has received increased attention in food packaging because of its excellent film-forming ability, biocompatibility, and intrinsic antimicrobial activity [7]. However, pure CS films have several practical limitations, including low mechanical strength, poor water vapor barrier properties, and insufficient long-term antimicrobial efficacy, which restrict their ability to control food spoilage during extended storage [8,9]. To overcome these limitations, incorporation of natural plant essential oils (EOs) into CS matrices has been widely explored as a strategy for enhancing antimicrobial and antioxidant functions [10]. Cinnamaldehyde (CIN), the major active component of cinnamon EO, exhibits broad-spectrum antibacterial activity against various foodborne pathogenic and spoilage bacteria [11]. Pomelo peel, a by-product widely generated in southern China, is often discarded, resulting in resource waste and environmental burden. However, it can be valorized into multiple products, including essential oil, pectin and dried peel preparations [12]. Pomelo peel essential oil (PPO) contains volatile compounds, such as limonene, and non-volatile oxygenated heterocyclic compounds, such as coumarins and furanocoumarins, which have been reported to exhibit good antibacterial and antioxidant activities in citrus essential oils [13,14].

Despite their potential, direct incorporation of EOs into hydrophilic CS films faces major obstacles, including poor water solubility, high volatility, and rapid loss of active components, which severely compromise the ability of the films to provide sustained control over microbial growth [15,16]. Several strategies have been proposed to overcome these obstacles, such as molecular encapsulation in cyclodextrins [17], pre-formed microcapsules and self-assembled nanorods [4,11], and electrospinning or Janus-type bilayer assembly [18,19]. However, all of these add one or more preparation steps to film fabrication, making the overall procedure comparatively complex. Pickering emulsion technology offers an effective solution to these challenges. Pickering emulsions are formed using solid particles (e.g., cellulose nanocrystals, protein nanoparticles) as stabilizers that irreversibly adsorb at the oil–water interface [20]. Consequently, these emulsions exhibit superior stability and low biotoxicity and can effectively encapsulate hydrophobic bioactive compounds, enabling their uniform dispersion and sustained release in hydrophilic polymer matrices [21,22]. Studies have confirmed that formulating EOs into Pickering emulsions can significantly improve their compatibility and sustained-release performance within polysaccharide films, thereby enhancing the long-term antimicrobial efficacy of the films [23,24].

Beyond interfacial stabilization, cellulose nanocrystals (CNCs) also serve a functional role within film matrices. For instance, in gum arabic/chitosan films, they enhance tensile strength and moisture resistance [25], while in whey protein/oregano essential oil films, the incorporation of 10 wt% grafted bacterial CNCs accelerates oil release via a porous morphology and boosts antibacterial activity to 80% [26]. These findings demonstrate that nanocellulose acts as both a reinforcing filler and a release regulator.

Despite these advances, most reported Pickering emulsion active films still rely on a single essential oil and require relatively high oil loadings (commonly 0.5–3%, *v*/*v*, of the film-forming solution) [20,27]. Moreover, the solid stabilizer is typically treated merely as an emulsifier, with its concurrent roles as a reinforcing filler and release regulator rarely being discussed.

In our previous work, a PPO/CIN Pickering emulsion applied directly as a strawberry coating inhibited four common foodborne bacteria at equal single-component concentrations, demonstrating a significantly enhanced effect [28]. However, this approach provided limited mechanical protection and necessitated a high oil dose, as the emulsion was not retained within a polymer matrix. To address these limitations, the present work incorporates this emulsion into a free-standing CS film, thereby substantially reducing the oil loading while introducing mechanical and barrier functionalities.

Based on these findings, we developed a CS-based composite film incorporating a Pickering emulsion co-loaded with PPO and CIN as a novel active food packaging strategy for controlling post-harvest spoilage of strawberries. Particular attention was paid to whether the co-loaded emulsion could lower the total oil content of the film to below 1% (*v*/*v*) while retaining antibacterial efficacy. Specifically, a composite stabilizer consisting of CS and C-CNCs was used to co-encapsulate PPO and CIN, simultaneously taking advantage of the fresh citrus aroma of PPO to mask the pungent odor of CIN and thereby improving sensory acceptability. The effects of the oil-to-water ratio on emulsion stability and the influence of emulsion loading on the microstructure, physicochemical properties (mechanical properties, water vapor barrier, optical properties, thermal stability), and functional performance of the composite film were systematically investigated. Finally, the effectiveness of the composite film in controlling fruit decay, weight loss, and softening was assessed through strawberry preservation experiments. This study provides a practical and sustainable active packaging solution for the food industry, contributing to the reduction in post-harvest losses and the improvement of food quality control.

## 2. Materials and Methods

### 2.1. Materials

Carboxylated cellulose nanocrystals aqueous dispersion (C-CNC, diameter: 20 nm, length: 200–500 nm, crystallinity: 74.6%, and surface functional group: carboxyl group) was purchased from Shenzhen Sailuna Technology Co., Ltd. (Foshan, China). CS (deacetylation degree: ≥95%, viscosity: 100–200 mPa·s, and formula weight: C_6n_H_11n_O_4n_, CAS: 9012-76-4) and CIN (purity: ≥98%, CAS: 104-55-2) were purchased from MACKLIN Biochemical Technology Co., Ltd. (Shanghai, China). PPO (purity: ≥98%) was purchased from Jiangxi Xinsen Natural Plant Essential Oil Co., Ltd. (Ji’an, China). Glacial acetic acid and glycerol were purchased from Xilong Scientific Co., Ltd. (Shantou, China). *Escherichia coli*, *Staphylococcus aureus*, *Listeria monocytogenes*, and *Vibrio parahaemolyticus* were maintained in the team’s laboratory. Fresh strawberries (variety Dandong 99) were purchased from the Hongxing Fruit Wholesale Market (Changsha, China). Fruits with intact appearance and free from mechanical damage or pests or diseases were selected.

### 2.2. Preparation of PPOEs

PPO/CIN composite emulsions (PPOEs) were prepared as described by Lu et al. [28,29], with slight modifications. Briefly, 0.16 g of CS was dissolved in 200 mL of 1% (*v*/*v*) acetic acid under stirring (DF-101S Constant-Temperature Magnetic Stirrer, Hunan Lichen Scientific Instrument Co., Ltd., Changsha, China) for 2 h to ensure complete dissolution. Thereafter, an equal volume of C-CNC solution (2.0%, *w*/*v*) was added, and the CS solution was stirred for 2 h. Subsequently, a mixture of PPO and CIN (volume ratio, 1:1) was added to the aqueous phase at oil-to-water ratios of 1:25, 1:50, 1:100, and 1:150 (*v*/*v*), respectively. The fresh composite suspensions were subjected to ultrasonic disruption using a JY92-2D ultrasonic cell disruptor (Ningbo Scientz Biotechnology Co., Ltd., Ningbo, China) operating at 200 W and 20 kHz for 20 min (2 s on, 2 s off) under ice-bath cooling. Finally, the crude emulsions were homogenized (10,000 rpm) using a T18 digital high-speed shearer (IKA-Works Inc., Staufen, Germany) for 6 min to obtain PPOEs. The PPOEs were stored at 4 °C until further use.

### 2.3. Characterization of PPOEs

#### 2.3.1. Morphology

A portion of each emulsion was transferred onto a microscope slide, covered with a coverslip, and observed using a FS-2A optical microscope (Leica Microsystems (Shanghai, China) Trading Co., Ltd., Shanghai, China) to characterize the droplet morphology and aggregation behavior. Before each measurement, the samples were vortexed to ensure uniform droplet dispersion.

#### 2.3.2. Particle Size, Zeta Potential, and Polydispersity Index

The average particle size, zeta potential, and polydispersity index (PDI) of the emulsions were measured using a Nano ZS 90 laser particle size analyzer (Malvern Instruments Ltd., Malvern, UK). Before each measurement, the samples were vortexed to ensure uniform droplet dispersion.

#### 2.3.3. Antimicrobial Activity

The antimicrobial activity of the emulsions against *E. coli*, *S. aureus*, *L. monocytogenes*, and *V. parahaemolyticus* was assessed using the Oxford cup method as described previously [30], with some modifications. Preactivated bacterial suspensions were standardized to OD_600_ = 0.1 using a spectrophotometer, and 50 µL of each bacterial suspension was added to 50 mL of medium that had been heated to a liquid state and cooled to approximately 50 °C. The mixture was evenly poured into a Petri dish. After the medium solidified, the Oxford cups were removed, and 100 μL of the sample solution was added to each well. The samples were incubated at 37 °C for 24 h, and the diameter of the inhibition zone was measured, with sterile distilled water used as the blank control. The emulsions (oil-to-water ratio of 1:50) were diluted with sterile distilled water to final emulsion concentrations of 20%, 40%, 60%, and 80% (*v*/*v*). Notably, the CIN concentration in the undiluted emulsion was only 0.98% (*v*/*v*), whereas the positive control consisted of 1.0% (*v*/*v*) CIN.

#### 2.3.4. Storage Stability

The freshly prepared emulsions were dispensed into transparent glass sample vials, and digital photographs were taken from days 1 to 21 to observe any stratification at room temperature.

### 2.4. Preparation of CS-PPOE Composite Films

**Figure 1:**
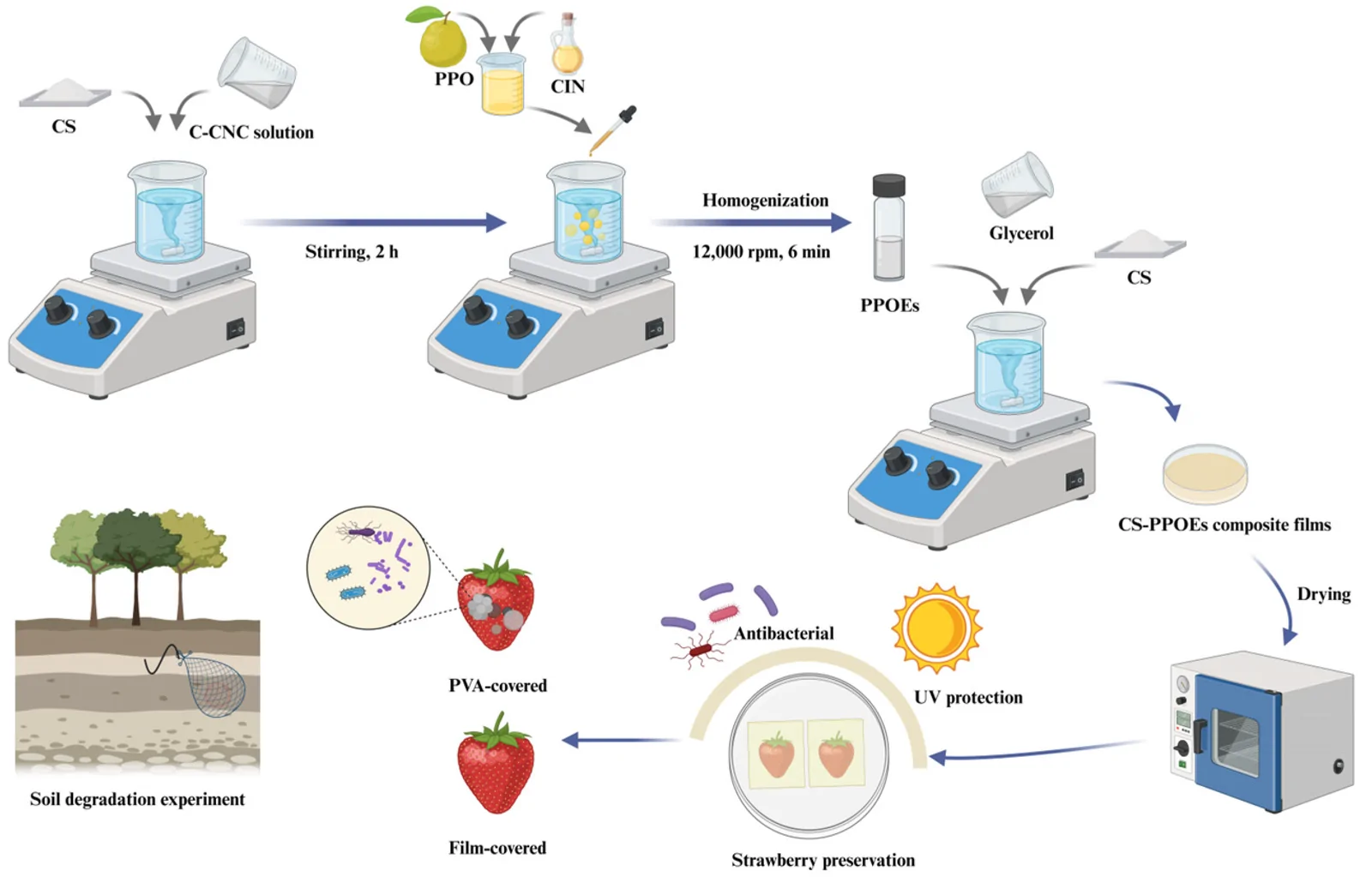
Schematic illustration of preparation of CS-PPOE films.

Chitosan (CS) was employed at two concentrations due to its distinct roles: as a co-stabilizer in the emulsion (0.08% *w*/*v*) and as the film-forming polymer (2% *w*/*v*). Based on emulsion characterization (morphology, particle size, zeta potential, and stability), the 1:50 oil-to-water emulsion was selected for film fabrication [31]. Briefly, CS (2 g) was dissolved in 100 mL of 1% (*v*/*v*) acetic acid at 37 °C for 2 h. After adding 3 mL of glycerol (plasticizer) and stirring for 30 min, varying volumes of PPOEs (0, 20, 30, 40, and 50 mL) were incorporated and stirred at room temperature for another 30 min. The resulting film-forming solutions (FFSs) were ultrasonicated (20 min) to remove air bubbles, and 240 mL of each was cast into 20 cm × 30 cm plastic plates, followed by drying at 37 °C for 32–48 h. The dried films were peeled and conditioned at 25 °C for 48 h before characterization. The films were designated CS, CS-20%PPOEs, CS-30%PPOEs, CS-40%PPOEs, and CS-50%PPOEs, corresponding to the volume (0, 20, 30, 40, and 50 mL) of PPOEs added per 100 mL of CS solution. These percentages denote the volume of the added PPOE emulsion and do not represent the essential oil content of the films. The overall preparation procedure of the CS-PPOE composite films is schematically illustrated in Figure 1.

### 2.5. Viscosity, Storage Modulus, and Loss Modulus of the Film Precursor Slurry

The rheological properties of the film precursor slurry were measured using a TA.XT Plus rheometer (Stable Micro Systems, Godalming, UK) equipped with a 40 mm parallel-plate setup. The viscosity, storage modulus (G′), and loss modulus (G″) of the film-forming precursor were measured in controlled shear rate mode (CR, 0.1–100 s^−1^) and controlled deformation mode (CD, 0.1–100 rad/s) at 25 °C.

### 2.6. Characterization of CS-PPOE Composite Films

#### 2.6.1. Fourier Transform Infrared Spectroscopy

The chemical structure of the composite films was examined using a Fourier transform infrared (FTIR) spectrometer (Thermo Nicolet Corporation, Madison, WI, USA) with KBr tablets. Measurements were recorded within the wavenumber range of 4000–400 cm^−1^, with 32 scans and a resolution of 4 cm^−1^.

#### 2.6.2. X-Ray Diffraction

The X-ray diffraction (XRD) patterns of the composite films were examined using a Rigaku SmartLab SE X-ray diffractometer (Rigaku Corporation, Tokyo, Japan), with a scanning range of 10–80° and a scanning rate of 0.02°/s.

#### 2.6.3. Thermogravimetric Analysis

The thermal stability of the composite films was measured using a TA Discovery TGA 550 thermal analyzer (TA Instruments, New Castle, DE, USA) under a nitrogen atmosphere. The samples were heated from 30 °C to 600 °C at a rate of 10 °C/min. Differential thermogravimetric (DTG) curves were derived from the first derivative of the thermogravimetric (TG) curves.

#### 2.6.4. Optical Characteristics and Color

The transmittance spectrum of the composite films was measured using a UV-2600 ultraviolet–visible spectrophotometer (Shimadzu, Corporation, Kyoto, Japan) over a wavelength range of 800–200 nm. The color properties (L* [lightness], a* [redness], and b* [yellowness]) of the films were measured using an UltraScan PRO automatic multifunctional colorimeter (HunterLab, Reston, VA, USA), which was calibrated against a standard white plate before the measurement.

$$\Delta E=\sqrt{{\left(\Delta L^{*}\right)}^{2}+{\left(\Delta a^{*}\right)}^{2}+{\left(\Delta b^{*}\right)}^{2}}$$

In this equation, ΔE is the color difference; ΔL*, Δa*, and Δb* are the differences in the corresponding color parameters between the standard white plate and each film sample.

#### 2.6.5. Scanning Electron Microscopy

The surface and cross-sectional morphological features of the composite films were characterized using a Sigma 300 scanning electron microscope (ZEISS AG, Oberkochen, Germany). The samples were placed in liquid nitrogen for embrittlement to obtain clean cross-sections.

#### 2.6.6. Water Contact Angle

The water contact angle (WCA) of the composite films was determined using a JY-82C surface tension tester (Chengde Dingsheng Testing Equipment Co., Ltd., Chengde, China). A 2 μL deionized water droplet was applied to the surface of 1 × 1 cm film samples, and the initial contact angle was immediately imaged and calculated.

#### 2.6.7. Thickness and Mechanical Properties

Film thickness was measured using a digital spiral micrometer (Sangliang, Tokyo, Japan) at 10 random points, of which three were randomly selected and averaged, with a 5 mm border area excluded to avoid edge effects. The mechanical properties (tensile strength [TS] and elongation at break [EAB]) of the composite films were evaluated using a TA.XT Plus texture analyzer (Stable Micro Systems, Godalming, UK). The samples were cut into rectangular strips 20 mm wide × 200 mm long and mounted between the tensile grips with an initial grip separation of 30 mm using a 5 kg load cell. The pre-test, test and post-test speeds were set to 1, 10 and 5 mm/s, respectively, the trigger force was 10 g, and the target mode was set to run until the specimen broke. Five strips were tested for each formulation.

#### 2.6.8. Water Vapor Permeability

The water vapor permeability (WVP) of the composite films was measured as described previously [32], with slight modifications. A test tube containing 3 g of anhydrous CaCl_2_ was hermetically sealed with a film sample and stored in a desiccator at 25 °C with 75% ± 2% relative humidity (RH). The weight of the test tube was recorded every 24 h for 7 days. WVP was calculated using the equation shown below.

$$\mathrm{WVP}=\frac{T\times \Delta M}{S\times \Delta P\times \Delta t}$$

In this equation, Δ*M* represents the weight change in the test tube (g), *T* represents the average thickness of the film (mm), Δ*P* represents the partial vapor pressure difference across the film (kPa), and *S* represents the effective test area of the film (m^2^) and Δt is the duration of the test (day; 7 d).

#### 2.6.9. Moisture Content and Water Solubility

The moisture content (MC) and water solubility (WS) of the composite films were measured on a dry basis as described previously [20]. Film samples (2 cm × 1 cm) were weighed (M_1_) and dried in an oven at 105 °C until a constant weight (M_2_) was achieved. The dried film samples were immersed in 30 mL of water for 24 h. The residual film samples were collected and dried again at 105 °C until a constant weight was achieved (M_3_). MC and WS were calculated using the formulas shown below.

$$\mathrm{MC}(\%)=\frac{M_{1}-M_{2}}{M_{1}}$$

$$\mathrm{WS}(\%)=\frac{M_{1}-M_{3}}{M_{1}}$$

#### 2.6.10. Strawberry Preservation

To evaluate the preservation performance of the composite films for perishable fruits, the films were used with commercial PVC film for simultaneous packaging for the same batch of strawberries with similar quality and size, which served as the control and is hereafter referred to as the CK group, since PVC cling film is the most widely used wrapping material for fresh-cut fruit trays and fruit boxes in retail settings such as fruit shops and supermarkets. The packaged strawberries were placed in Petri dishes and stored indoors (27 ± 4 °C). Visual changes were observed over time and photographed daily. To assess changes in the quality of strawberries during storage, decay rate, weight loss rate, hardness, and the total soluble solids (TSS) content were measured. The hardness of the strawberries was measured using a GY-3 fruit hardness tester (Aipu Metrology Co., Ltd., Quzhou, China), and TSS content was measured using an M15194 handheld refractometer (Beijing Haifuda Technology Co., Ltd., Beijing, China).

#### 2.6.11. Biodegradability

To evaluate the biodegradability of the composite films, samples were buried in natural soil at a depth of approximately 5 cm for 21 days, together with the commercial PVC film (CK) used as the non-degradable reference material, and their morphological changes were monitored periodically.

### 2.7. Statistical Analysis

One-way analysis of variance (ANOVA) was performed using SPSS (version 26.0; IBM, Armonk, NY, USA). Duncan’s multiple range test was used for post hoc comparisons of the means. Differences were considered statistically significant at *p* < 0.05. All experiments were performed with at least three independent replicates (*n* ≥ 3) and the results are expressed as mean ± standard deviation (SD). Five strips were tested per film for the tensile measurements and ten randomly selected points per film for the color measurements; all other determinations were carried out in triplicate.

## 3. Results and Discussion

### 3.1. Effect of the Oil-to-Water Ratio on Emulsions

**Figure 2:**
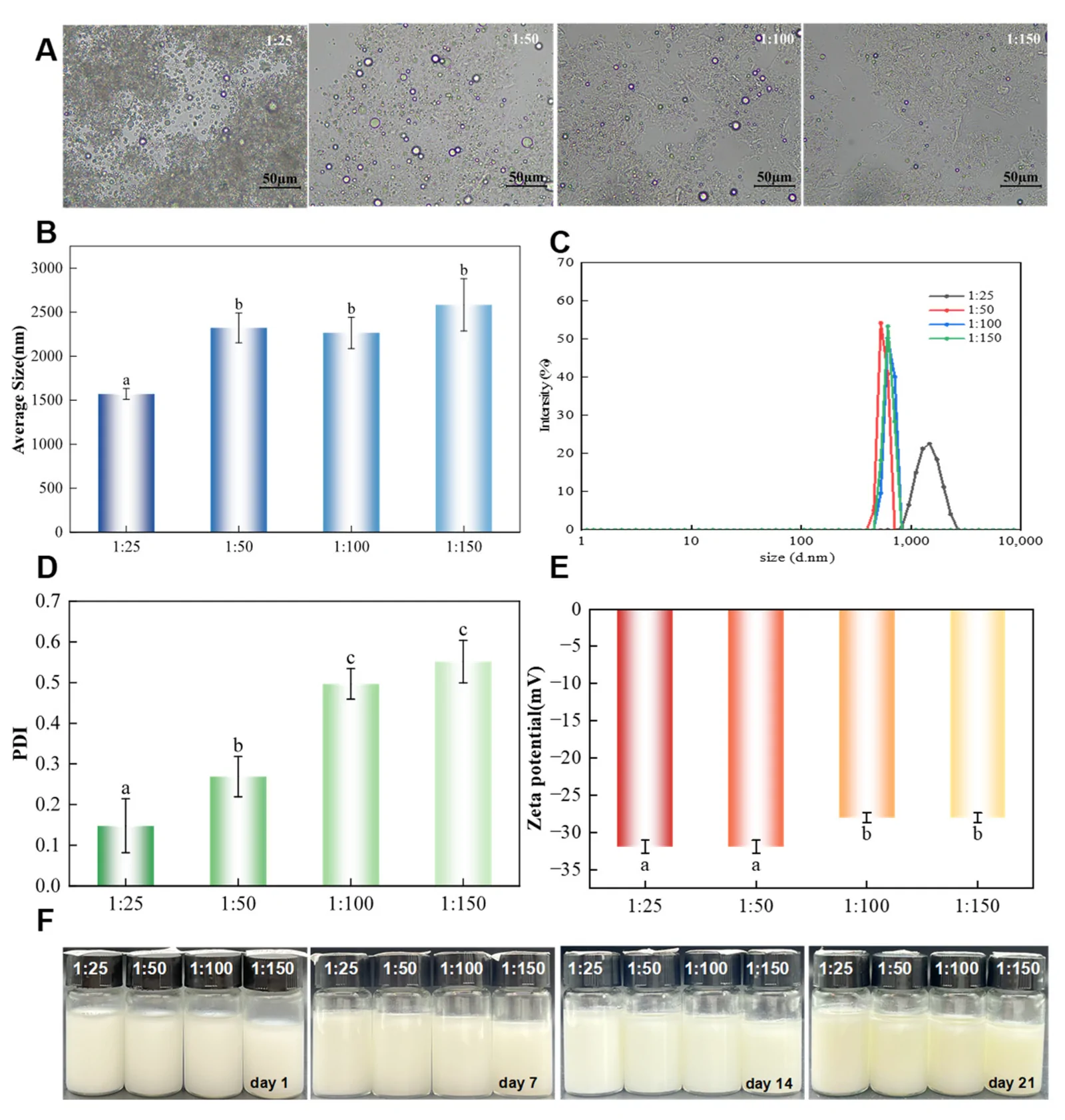
Effect of the oil-to-water ratios (*v*/*v*) on the properties of the PPOEs. Optical micrographs (**A**), average size (**B**), intensity-based size distribution (**C**), polydispersity index (PDI, (**D**)), zeta potential (**E**), and appearance of emulsions (**F**). Different lowercase letters above the bars indicate significant differences (*p* < 0.05).

Particle size and distribution are important factors for evaluating system stability [29], as their changes reflect dispersion uniformity and anti-aggregation ability. Figure 2A shows the optical micrographs of emulsions with 1% C-CNC at different oil-to-water ratios. At a high oil-to-water ratio (1:25), droplet accumulation and aggregation were evident in the micrographs of the undiluted emulsions, which can be attributed to the combined effect of high viscosity and insufficient interfacial coverage: the increased viscosity hindered shear-induced droplet refinement [33], while the fixed amount of C-CNCs could not fully cover the greatly enlarged oil–water interface, so insufficiently stabilized droplets bridged and flocculated into large clusters. In contrast, at a low oil-to-water ratio (1:100), progressive phase separation occurred, consistent with appearance changes after 21 days of storage (Figure 2F). This finding can be attributed to insufficient oil–water interface area, where emulsifiers cannot be fully adsorbed, leading to a weak interfacial film [34]. A balanced ratio (1:50) resulted in uniform droplet distribution without aggregation, indicating superior stability.

Figure 2B–D show the average particle size, intensity, and PDI of PPOEs as a function of oil-to-water ratio. The average droplet size decreased with increasing oil phase proportion, reaching a minimum of 1572.67 ± 63.06 nm at a ratio of 1:25 (*v*/*v*). PDI also achieved the lowest value (0.148 ± 0.066) at this ratio. These values were significantly lower than those of other samples (*p* < 0.05). It should be noted that the droplet sizes reported here were measured on emulsions diluted 1:1000 (*v*/*v*) with ultrapure water immediately before DLS analysis; dilution and vortexing disrupt the weakly flocculated clusters, so the DLS values reflect the size of the primary droplets rather than the flocs visible in the micrographs of the undiluted emulsions. The coexistence of small primary droplets and visible flocculation at the native concentration therefore explains the apparent contradiction between the small DLS-derived size and the microscopic observation of aggregation at 1:25. PDI, ranging from 0 to one, indicates the homogeneity of particle size distribution, with lower values reflecting better dispersion uniformity [35].

Zeta potential is another key indicator of emulsion stability, with larger absolute values indicating better stability owing to enhanced electrostatic repulsion that resists flocculation. A zeta potential value exceeding 30 mV indicates effective inhibition of droplet coalescence, which improves system stability [36]. As shown in Figure 2E, all samples were negatively charged. The 1:25 and 1:50 groups exhibited absolute zeta potential values of >30 mV, confirming their superior stability. These findings were consistent with the particle size distribution (Figure 2C), wherein a rightward shift in the peak with decreasing oil phase proportion indicated larger droplets. This trend aligned with the progressive phase separation observed after storage (Figure 2F).

Overall, the optimized formulation with an oil-to-water ratio of 1:50 exhibited excellent storage stability and uniform, small droplet sizes, making it an ideal candidate for subsequent preparation of composite films.

### 3.2. Antimicrobial Effect of the Emulsions

**Figure 3:**
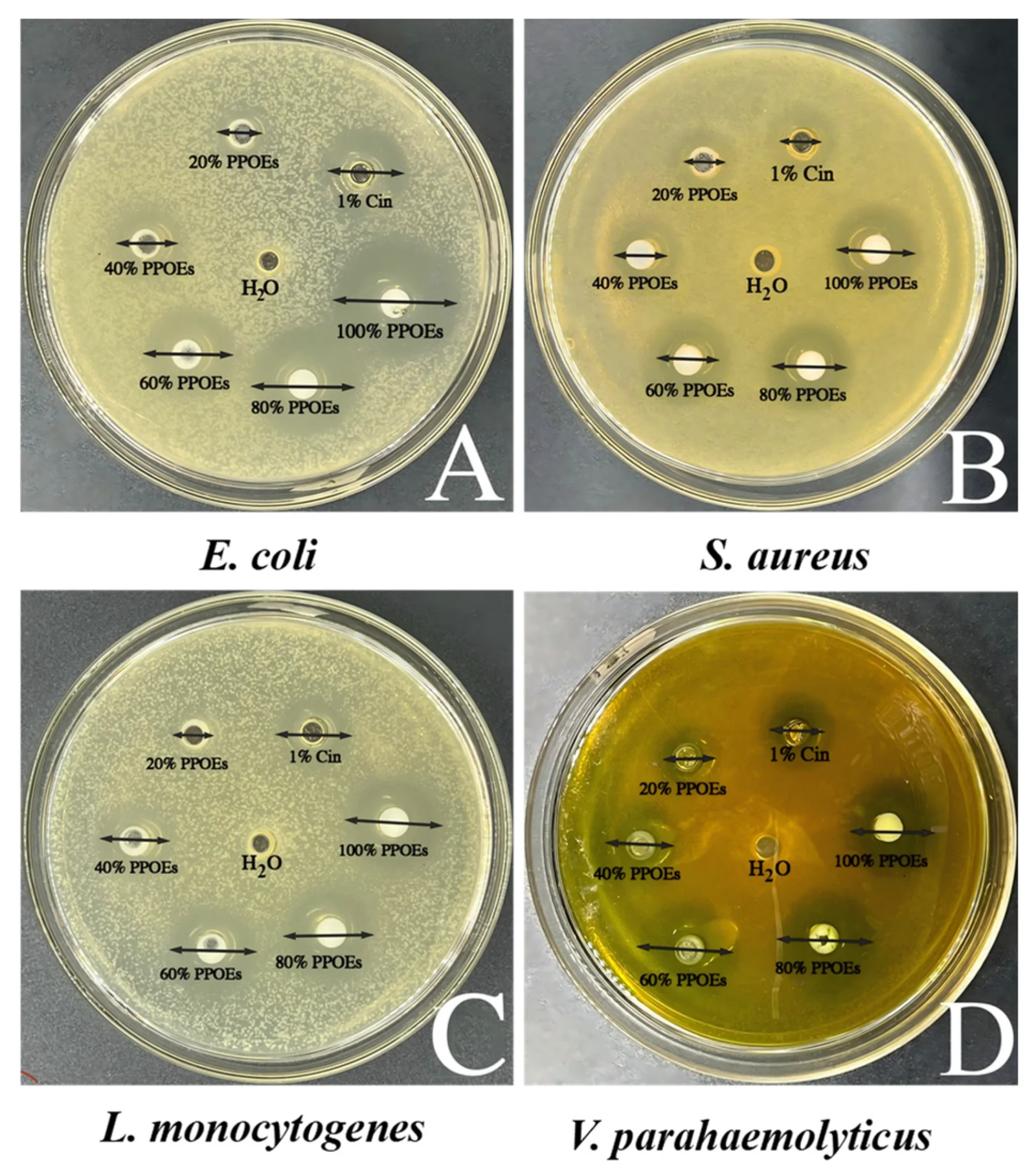
Inhibition zones of PPOEs at different concentrations against *E. coli* (**A**), *S. aureus* (**B**), *L. monocytogenes* (**C**), and *V. parahaemolyticus* (**D**). H_2_O, sterile distilled water (negative control); 1% CIN, 1% (*v*/*v*) cinnamaldehyde (positive control); 20–100% PPOEs, PPOEs diluted to the indicated volume fraction (*v*/*v*) with sterile distilled water.

The antimicrobial efficacy of the PPOEs was significantly higher than corresponding single-component treatments, consistent with our previous work [28]. Specifically, the antimicrobial activity of 20% PPOEs was comparable to that of 1% pure CIN against *L. monocytogenes* and *V. parahaemolyticus*, whereas 40% and 60% PPOEs matched the efficacy of 1% CIN against *S. aureus* and *E. coli*, respectively. Most notably, 100% PPOEs achieved an exceptional inhibition zone of 32.98 ± 0.12 mm against *E. coli*. These results indicate that the combination of PPO and CIN significantly enhances the antimicrobial performance of each component. This significantly enhanced effect may be attributed to naringin and cinnamic acid (loaded in PPOEs) inhibiting microbial proliferation. The antimicrobial potency of PPOEs is significantly superior to that of single-component EO delivery systems reported in recent studies [37]. Notably, 20% PPOEs contained only 0.196% (*v*/*v*) CIN, about one fifth of the concentration in the 1% CIN control, yet produced inhibition zones against L. monocytogenes (12.75 ± 0.50 mm) and V. parahaemolyticus (13.17 ± 0.43 mm) comparable to those of the free CIN control (12.26 ± 0.62 and 13.24 ± 0.20 mm). Emulsification therefore markedly increases the apparent antibacterial efficiency of CIN. The inhibition zones obtained at different PPOE concentrations are shown in Figure 3, and the corresponding diameters are summarized in Table 1.

### 3.3. Structural Characterization of Composite Films

#### 3.3.1. Rheological Properties of the Film Precursor Slurry

**Figure 4:**
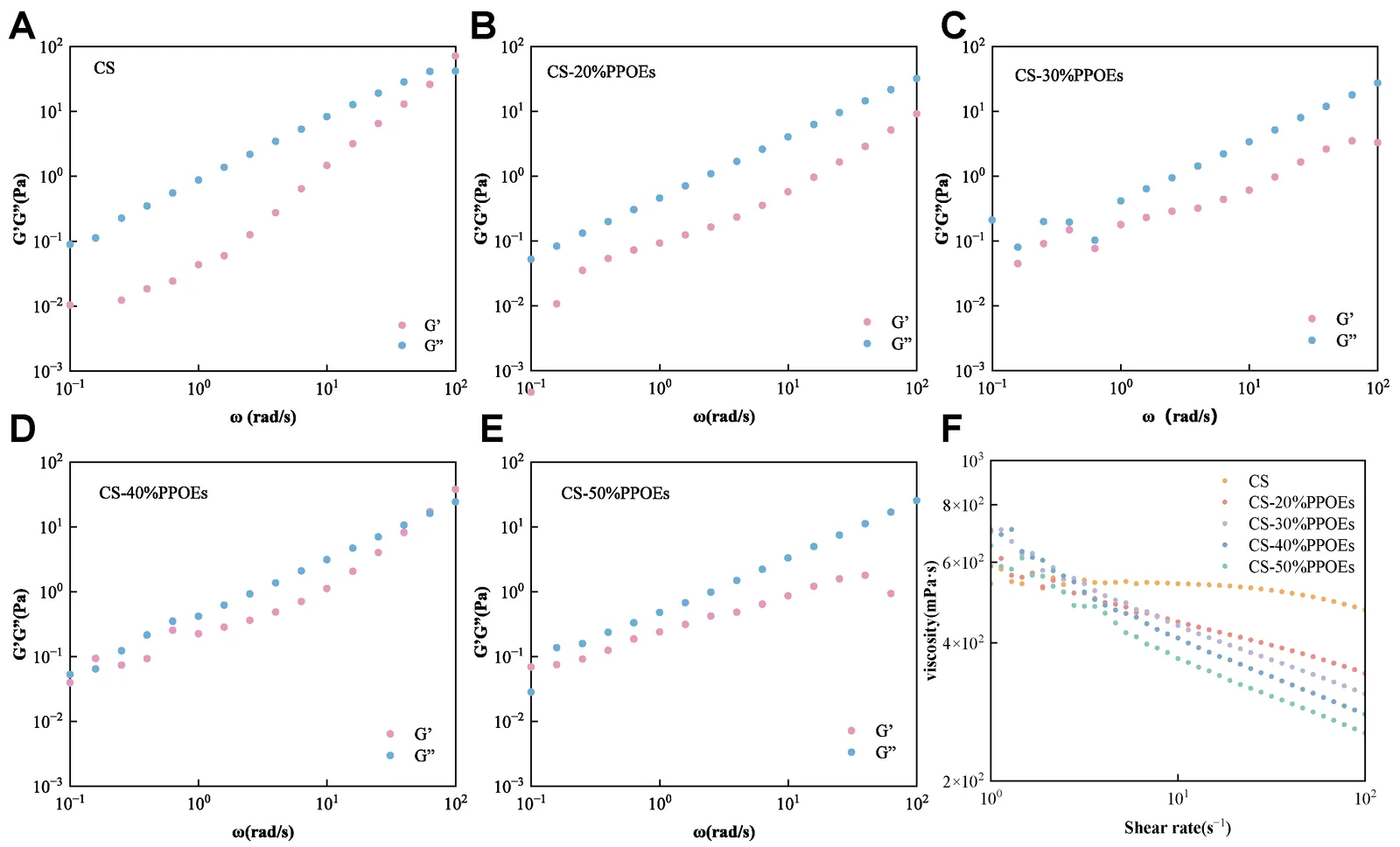
Rheological properties and structural characteristics of the composite films. (**A**–**F**) Storage modulus (G′), loss modulus (G″), and viscosity of precursor slurries. Measurements were made at 25 °C.

Rheological analysis (Figure 4A–F) revealed that all samples exhibited liquid-like viscoelastic behavior (G″ > G′) without a sol–gel transition or a distinct gel transition point, consistent with polysaccharide-based FFSs [31].

The G′ and G″ of the composite films increased with angular frequency, indicating their clear frequency-dependent viscoelastic behavior and structural integrity under dynamic oscillation [31,38]. With the addition of PPOEs, the difference between G′ and G″ gradually decreased, primarily owing to an increase in G′. These findings suggest that PPOEs interact with CS through hydrogen bonding, ionic bonding, and hydrophobic interactions, thereby enhancing the elasticity of the system [31].

In terms of viscosity, all samples exhibited shear-thinning (pseudoplastic) behavior: viscosity decreased continuously with increasing shear rate, a characteristic of a non-Newtonian pseudoplastic fluid. This property is critical for ensuring uniformity in the film-forming process [39]. This optimized rheological profile enhances spreadability and ensures thickness uniformity during film casting.

#### 3.3.2. FTIR Spectroscopy

**Figure 5:**
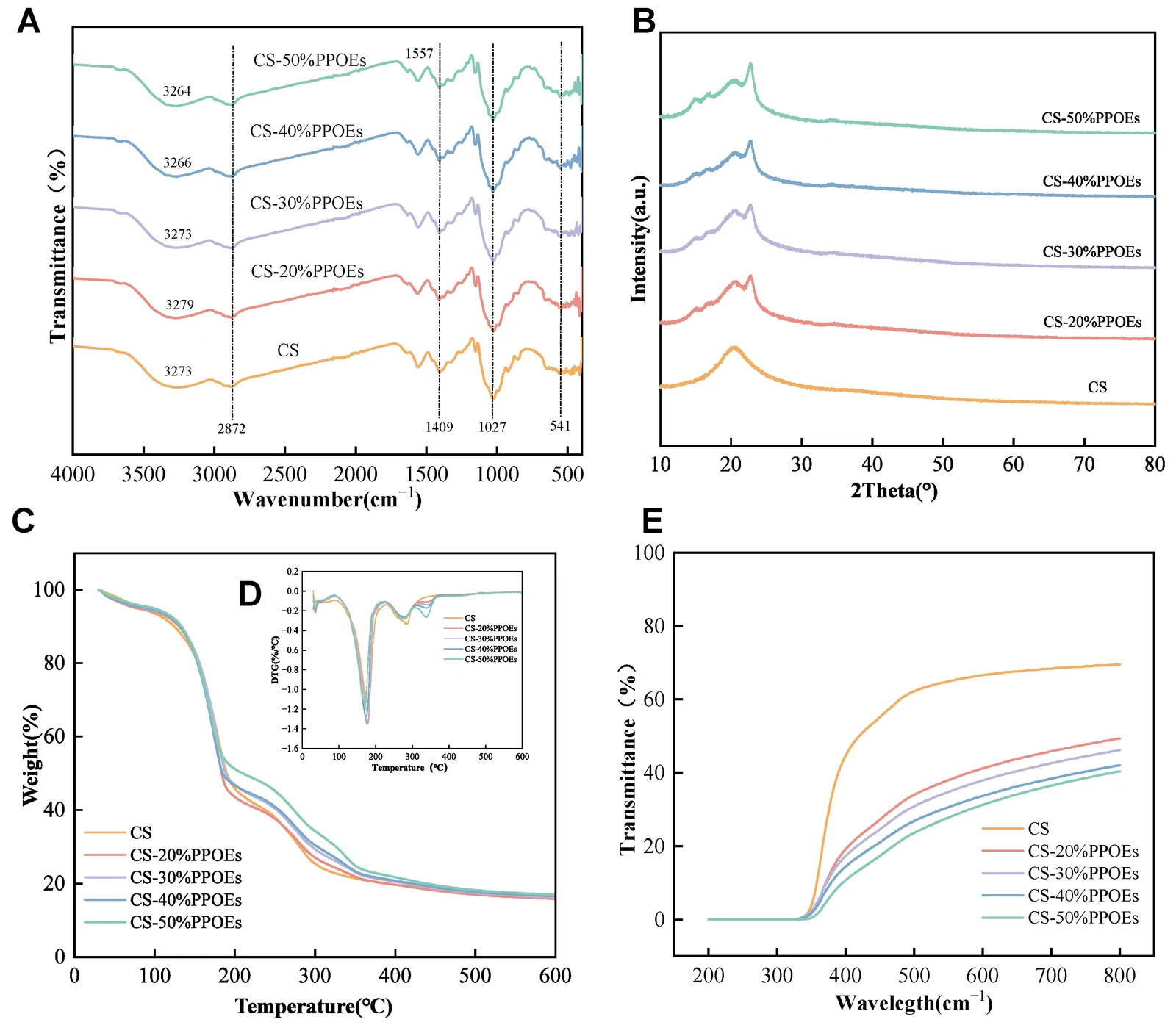
Fourier transform infrared spectra (FTIR, (**A**)), X-ray diffraction patterns (XRD, (**B**)), thermogravimetric analysis thermograms (TGA, (**C**)), differential thermogravimetric curves (DTG, (**D**)), and UV resistance (**E**) of composite films.

Figure 5A shows the FTIR spectra of composite films with different PPOE loadings. The broad, intense absorption band at 3273 cm^−1^ is attributed to the intra- and intermolecular O–H and N–H stretching vibrations of CS, reflecting the inherent hydrogen bond network in the CS matrix [25,40]. This stretching peak exhibits a non-monotonic shift. Compared with the pure CS film (3273 cm^−1^), the CS film with 20% PPOEs showed a slight blue shift to 3279 cm^−1^, indicating that a low concentration of PPOEs disrupts the original hydrogen bond network and weakens hydrogen bond interaction, consistent with the findings of a previous study [40]. When the PPOE content was increased to 30%, the peak returned to 3273 cm^−1^, identical to that of the pure CS film. This non-monotonic behavior indicates that two opposing effects coexist at this loading: the disruption of the original CS–CS hydrogen bonds (blue-shift contribution) and the formation of new PPOE-CS hydrogen bonds (red-shift contribution) counterbalance each other, so the apparent peak position remains unchanged even though the network is being reorganized. When PPOE content was increased to 40% and 50%, the peak showed a red shift to 3266 and 3264 cm^−1^, respectively, indicating the formation of a stronger and denser hydrogen bond network [41]. These findings may be attributed to intermolecular interactions between the abundant phenolic hydroxyl and carbonyl groups in PPOEs and the hydroxyl and amino groups in CS, consistent with molecular dynamics simulations showing that active components enhance hydrogen bond interaction and reduce binding energy [34]. The introduction of PPOEs did not significantly alter the characteristic peaks of the composite film, inducing only minor intensity changes and slight shifts (e.g., the C–O–C stretching peak at 1027 cm^−1^ intensified with increasing PPOE content), indicating that PPOEs exhibited good compatibility and uniform dispersion within the film matrix. This interpretation is consistent with the rheological behaviour described in Section 3.3.1, where the increase in G′ and the narrowing of the G′–G″ gap pointed to the formation of a more elastic network through the same set of interactions.

#### 3.3.3. XRD Patterns

XRD was used to assess the incorporation of components and evaluate their impact on the crystal structure of the films. As shown in Figure 5B, the pure CS film exhibited a broad diffraction peak at 2θ ≈ 20°, indicating a semi-crystalline/amorphous structure typical of polysaccharide-based films [20,42].

With the introduction of PPOEs, the diffraction peaks gradually became sharper and their intensity significantly increased. Moreover, as the amount of PPOEs added increased, the peaks became sharper, indicating that the crystallinity of the system improved and molecular chains became more ordered after PPOE addition [20,43]. The consistency in the positions of the characteristic diffraction peaks eliminated the possibility of the formation of new crystalline phases, whereas the absence of independent characteristic peaks for PPOEs further confirmed the molecular-level compatibility between PPOEs and CS, indicating that the two components formed a homogeneous blend system.

#### 3.3.4. TG Analysis

TG analysis was performed to evaluate the thermal stability of the composite films. As shown in Figure 5C, all films exhibited a typical multi-stage thermal decomposition profile within the test range (30–600 °C), consistent with previous reports on polysaccharide-based films [41]. The first stage (30–130 °C) is primarily attributed to the evaporation of free and bound water in the film [43]. The second stage (130–320 °C) corresponds to the thermal degradation of thermally unstable active substances such as EOs and represents the primary stage of mass loss [44]. The third stage (320–600 °C) results from the oxidative degradation and thermal decomposition of the CS polymer backbone (including the cleavage, dehydration, and carbonization of polysaccharide chains) [45]. The thermal degradation trends of all composite films were generally consistent. Compared with the pure CS film, CS-PPOE composite films exhibited higher mass retention rates within the 180–320 °C range, and the mass retention rate at the same temperature gradually increased with increasing PPOE content, indicating that the introduction of PPOEs effectively delayed the thermal decomposition process of the CS matrix.

The DTG curve (Figure 5D) reflected the variation in thermal degradation with temperature. Compared with the T_max_ of the pure CS film, that of CS-PPOE composite films shifted slightly toward higher temperatures as PPOE content was increased, indicating the enhanced thermal stability of the composite films. This improvement can be attributed to the formation of stabilizing interactions, such as hydrogen bonds, between PPOEs and CS molecules, which effectively restrict the movement of polymer chains. Furthermore, the phenolic compounds in EOs promote the formation of a more stable char layer at high temperatures, further enhancing the residual char yield of the composite film [19]. The incorporation of PPOEs creates a dense composite network structure with strong hydrogen bond interactions, which improves thermal stability. This structure enables the film to better withstand heat treatment or heat-sealing processes during food packaging, providing a crucial guarantee for its practical application.

#### 3.3.5. Light Transmittance (UV Resistance), Thickness, and Color

UV irradiation can induce lipid oxidation, nutrient degradation, and sensory properties in food. UV resistance is an important functional factor in food packaging [32]. As shown in Figure 5E, the pure CS film exhibited high transmittance in the UV range (400–200 nm), indicating low UV resistance. However, with the addition of PPOEs, the UV transmittance of the composite film decreased significantly and continued to decrease as PPOE content was increased, suggesting that UV resistance increases with increasing PPOE content. This effect is attributable to the strong UV absorption of terpenoids present in PPO [46].

The pure CS film showed the highest L* value, indicating the highest transparency. However, the addition of PPOEs reduced the L* value and increased the a* and b* values, indicating decreased brightness, and enhanced redness and yellowness of the CS film. Consistent with this, the total color difference (ΔE) relative to the white standard plate increased from 51.32 ± 0.13 to 58.48 ± 0.34, indicating a progressively greater color deviation from white with increasing PPOE loading (Table 2).

#### 3.3.6. SEM

**Figure 6:**
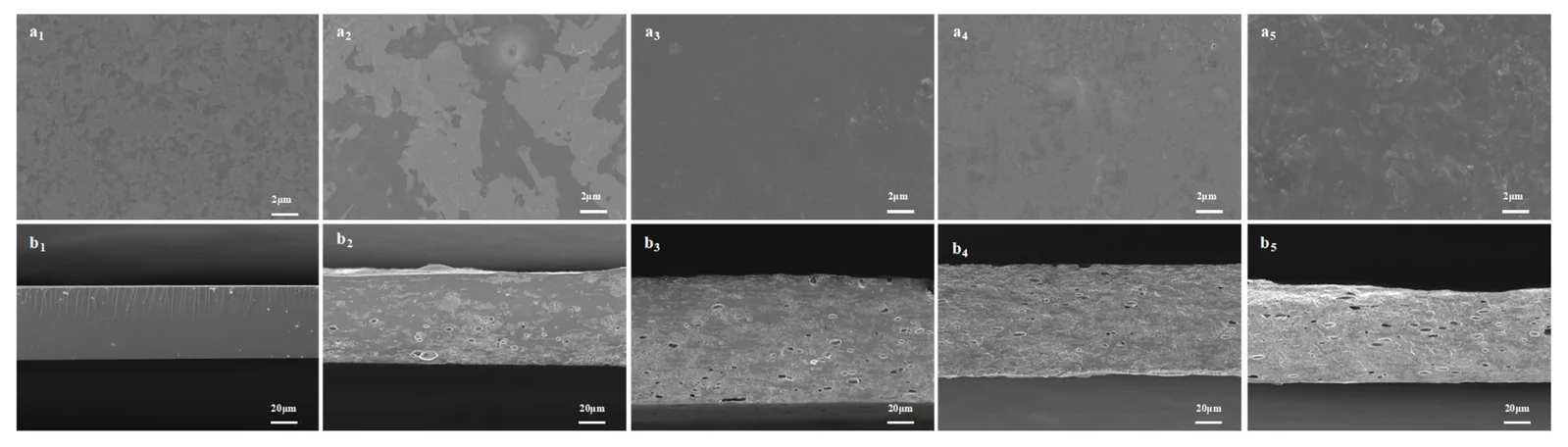
Scanning electron microscopy (SEM) images of the surface (**a_1_**–**a_5_**) and cross-section (**b_1_**–**b_5_**) of the composite films.

Microscopic images of the film surface and cross-section were obtained using scanning electron microscopy (SEM). As shown in Figure 6, the CS film exhibited a smooth surface and a dense, homogeneous cross-sectional structure. After the incorporation of PPOEs, the cross-sectional morphology of the film gradually transitioned toward increased roughness, characterized by the emergence of protrusions and micropores. Although the CS-30%PPOE film maintained a relatively smooth surface, its cross-section showed a well-developed microporous structure and irregular edges, suggesting the formation of a sophisticated pore network. Conversely, evident oil droplet aggregation was observed on the surface of the CS-40%PPOEs and CS-50%PPOEs films. Their corresponding cross-sections revealed a stratified pore architecture and distinct phase separation, likely attributable to the excessive accumulation or agglomeration of free EO within the polymer matrix [17]. These findings indicate that a PPOE content of 30–40% is optimal, as it facilitates effective integration into the CS matrix with minimal disruption to its structural orientation. It should be noted that the C-CNCs entered the film together with the emulsion droplets rather than being added separately, and the present design does not allow their individual contribution to the features described above to be isolated. A contribution of C-CNCs is nevertheless likely, since nanocellulose acts in polysaccharide films both as a reinforcing filler and as a regulator of oil release [25,26], and such particles are themselves known to promote roughness and porosity in the dried matrix. The morphological changes observed here are therefore most reasonably ascribed to the combined action of the emulsified oil phase and the nanocrystals carried with it.

#### 3.3.7. WCA

**Figure 7:**
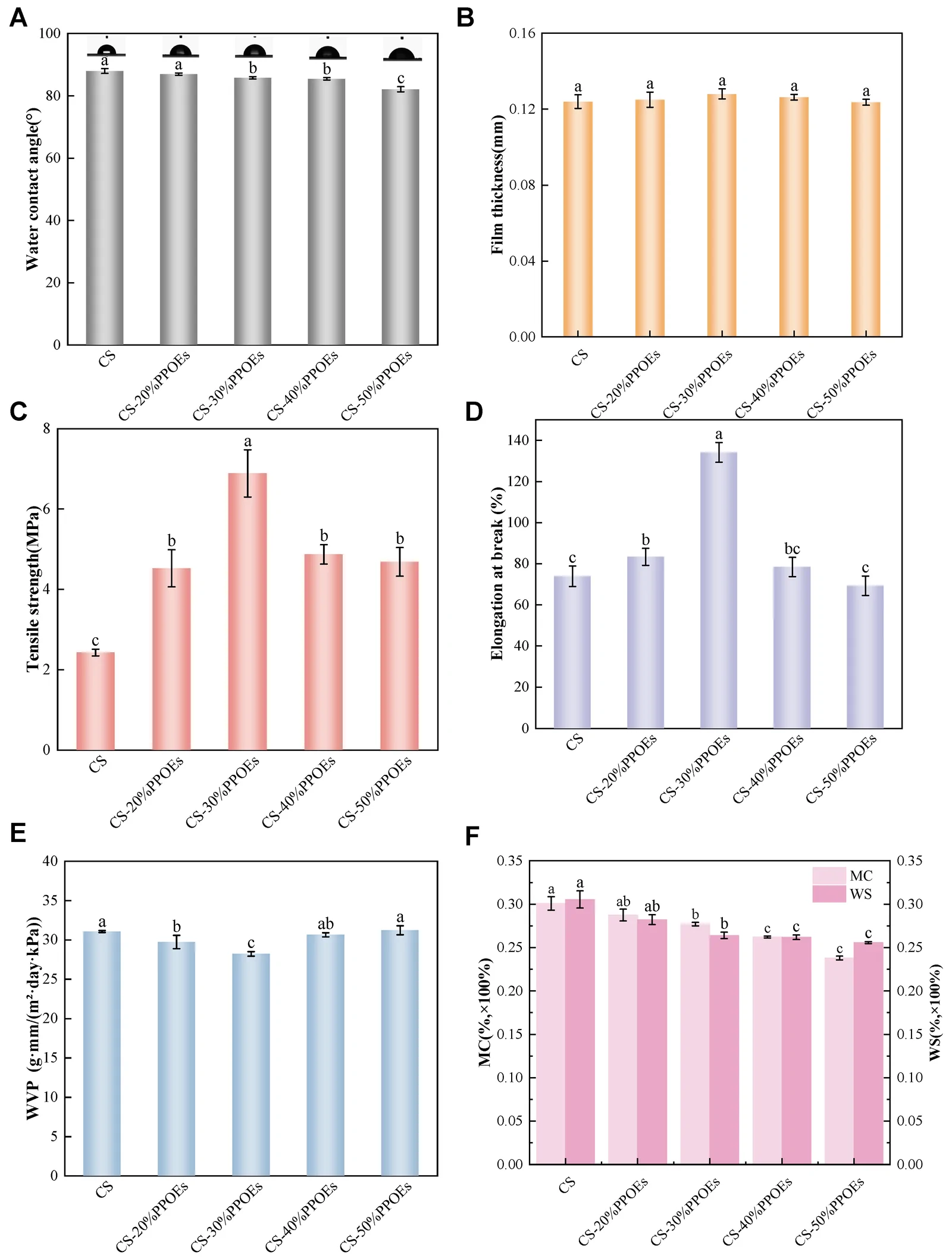
Water contact angle (WCA, (**A**)), film thickness (**B**), tensile strength (TS, (**C**)), elongation at break (EAB, (**D**), water vapor permeability (WVP, (**E**)), moisture content (MC, (**F**)), and water solubility (WS, (**F**)). Different letters indicate a significant difference (*p* < 0.05).

WCA is a critical indicator for evaluating the hydrophilic and hydrophobic properties of a film surface. As shown in Figure 7A, all films exhibited WCA values exceeding 65°, indicating hydrophobic behavior. However, with increasing PPOE content, the contact angle decreased, suggesting an increase in hydrophilicity. This decrease may occur because excess emulsion droplets may accumulate within the film, disrupting its dense and continuous structure and thereby reducing its hydrophobicity [44].

#### 3.3.8. Film Thickness and Mechanical Properties

The physical and mechanical properties of the composite films are summarized in Figure 7B–D. As shown in Figure 7B, the thickness of the composite films ranged from 0.124 ± 0.002 to 0.128 ± 0.003 mm and showed no significant difference among formulations (*p* > 0.05), as indicated by the identical letters (a). Mechanical properties were evaluated based on TS (Figure 7C) and EAB (Figure 7D). TS initially increased with increasing PPOE content, reaching a maximum at 30% PPOEs before gradually decreasing. This initial increase is primarily ascribed to the robust interfacial interactions, specifically hydrogen bonding between PPOEs and the CS matrix. These interactions effectively occupy the free volume within the polymer chains and enhance the cross-linking density of the composite network. However, a further increase in PPOE content (>30%) reduced TS. This decrease is likely attributed to the agglomeration of excess PPOEs in the matrix, leading to local stress concentrations and defects that disrupt the polymer’s continuity [27].

Changes in EAB showed a similar trend. Compared with the pure CS film, CS-PPOE films exhibited an initial increase followed by a subsequent decrease in EAB. This finding indicates that the incorporation of PPOEs enhances the flexibility and ductility of the composite films. This improvement can be attributed to the plasticizing effect of active EOs in PPOEs, which weakens the strong intermolecular interactions between CS chains. By increasing the mobility of the molecular chains and reducing film brittleness, PPOE allows the matrix to undergo greater deformation under stress without breaking easily [44]. Because the C-CNCs were introduced together with the emulsion, part of the reinforcement may also be ascribed to the nanocrystals rather than to the oil phase alone.

#### 3.3.9. WVP

WVP is a critical indicator of a packaging material’s ability to block water vapor [47]. It quantifies the rate at which water vapor molecules diffuse from the high-humidity side (such as the food interior) to the low-humidity side (external environment) [48]. An ideal food packaging material should exhibit an appropriate WVP [49]: sufficiently low to prevent moisture loss from fresh produce (thus preserving weight, texture, and freshness) but not so low as to cause internal condensation, which may promote microbial growth (such as bacteria and mold).

As shown in Figure 7E, the WVP of the composite films initially decreased and subsequently increased with increasing PPOE content. The pure CS film exhibited the highest WVP value, reflecting its strong hydrophilicity and limited water vapor barrier capability. The addition of PPOEs significantly reduced WVP, indicating that the interaction between PPOEs and the CS matrix improved barrier properties. However, excessive PPOE loading led to a decline in the water vapor barrier performance. This decline can be attributed to the agglomeration of excess additives within the polymer matrix, which disrupts the originally dense network structure and creates micropores and cracks within or on the film surface [27].

#### 3.3.10. MC and WS

As shown in Figure 7F, as PPOE content was increased from 20% to 50%, both the MC and WS of the composite films exhibited a significant downward trend (*p* < 0.05), indicating that the introduction of PPOEs effectively enhances the water resistance and structural stability of the CS matrix. This improvement can be attributed to three factors. First, the hydrophobic components of PPOEs (such as EOs) occupy the hydrophilic binding sites between CS molecular chains and physically block the binding of water molecules to the hydrophilic groups of CS (such as –OH and –NH_2_), thereby reducing water absorption and retention [32,50]. Second, strong intermolecular hydrogen bonding and electrostatic interactions between PPOEs and CS construct a more compact network structure, restricting the movement of CS chains and hindering water penetration and diffusion, which significantly reduces the film’s solubility in water [20]. Finally, the uniform dispersion of PPOEs within the CS matrix fills microscopic pores and further reduces water storage space and swelling through a densification effect [31]. Overall, the addition of PPOEs significantly increases the water resistance of CS-PPOE composite films through multiple mechanisms, including hydrophobic modification, enhanced intermolecular interactions, and structural densification. This increase is advantageous for their application in high-humidity food packaging.

#### 3.3.11. Freshness Retention Properties of Composite Films

**Figure 8:**
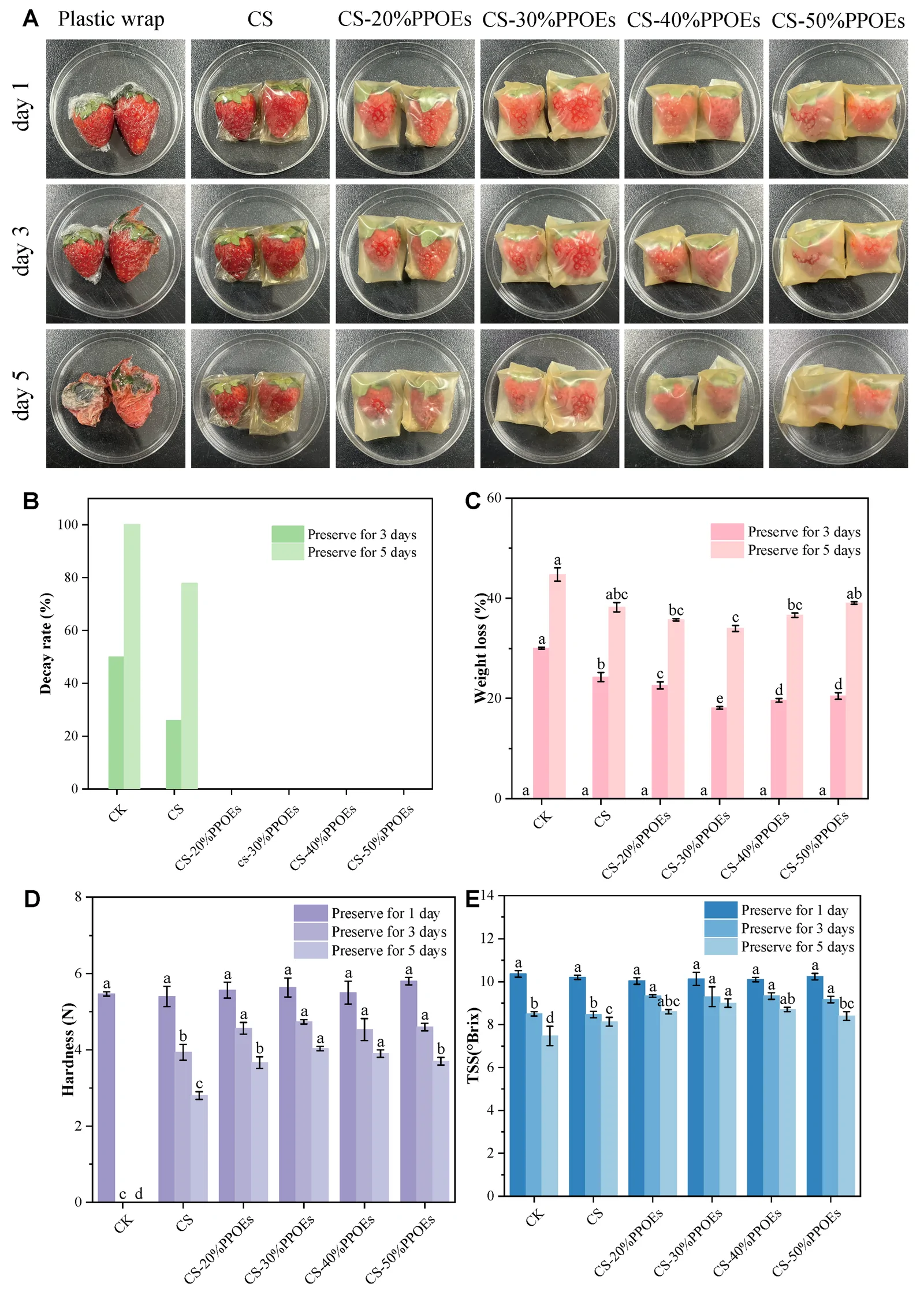
Fruit preservation properties of composite films. Digital photo of packaged strawberries (**A**), decay rate (**B**), weight loss (**C**), hardness (**D**), and the total soluble solid (TSS) content (**E**) in strawberries. Different letters indicate a significant difference among the treatment groups on the same day (*p* < 0.05). Strawberries were stored at 27 ± 4 °C.

As a typical berry with high post-harvest activity, strawberries exhibit vigorous metabolism and are highly susceptible to microbial infection. Consequently, they have an extremely short shelf life at room temperature, which severely limits their distribution and sales. In this study, we systematically evaluated the efficacy of CS-PPOE composite films in strawberry preservation. As shown in Figure 8A,B, significant differences were found in appearance and decay rate among the different treatment groups (*p* < 0.05). In the CK group (PVC film), water droplets were observed on the surface of strawberries during storage, and by day 5, strawberries had completely molded and rotted, with a decay rate of 100%. This decay is likely attributed to the poor barrier properties of the packaging, which created high humidity in the sealed environment, providing favorable conditions for microbial growth and causing the strawberries to soften and spoil more easily. In contrast, strawberries in the pure CS film treatment group retained their intact shape after 5 days, exhibiting significantly better preservation than the CK group (PVC film). The performance of CS-PPOE composite films was more significant: All fruits retained a bright color, and the decay rate was 0%. This inhibitory effect is superior to that of similar active films under ambient storage conditions [44]. The anti-rot effect of the CS-PPOE composite film can be attributed to the significantly enhanced action of the active ingredients in PPOEs, consistent with the results of the antibacterial experiments.

Weight loss rate, primarily caused by transpiration and respiratory metabolism, is a critical indicator of post-harvest water loss and freshness in fruits and vegetables. As shown in Figure 8C, the weight loss rate of strawberries in the CK group (PVC film) increased rapidly with storage time, reaching nearly 45% after 5 days, accompanied by juice exudation. Although strawberries packaged in composite films also experienced some weight loss, the addition of PPOEs reduced the weight loss rate, with the lowest rate observed at 30% PPOEs. The formation of a denser, more ordered microstructure significantly reduced WVP, thereby effectively blocking water exchange between the fruit and the external environment. Furthermore, the strong antibacterial activity of the composite film inhibited microbial activity, which indirectly reduced juice exudation caused by cell membrane damage.

Hardness is a critical parameter for evaluating the texture, freshness, and shelf life of fruits. The post-harvest decline in strawberry firmness is primarily driven by the activation of cell-wall-degrading enzymes, pectin degradation, and moisture loss. As shown in Figure 8D, the firmness of the CK group (PVC film) decreased rapidly during storage. Although the pure CS film effectively mitigated this decline, CS-PPOE composite films exhibited superior performance (*p* < 0.05). Notably, formulations incorporating 30% and 40% PPOEs yielded better results, significantly delaying fruit softening.

Fruit softening is primarily associated with the degradation of cell wall structure and the loss of film integrity. Studies reported that bioactive materials can inhibit the activity of cell-wall-degrading enzymes by regulating the expression of metabolism-related genes [51]. Furthermore, polyphenol-rich extracts can retard ripening and softening by suppressing oxidative processes [49]. Consequently, the protective effect of the CS-PPOE composite film on fruit firmness can be attributed to two main mechanisms: first, the phenolic components in PPOEs inhibit the activity of cell-wall-degrading enzymes (such as pectinases and cellulases), thereby preserving the structural integrity of the cell wall; second, these active compounds reduce film lipid peroxidation caused by reactive oxygen species (ROS), maintaining cell film integrity and consequently delaying fruit softening.

TSS content is a critical indicator of fruit flavor and ripeness. It tends to decrease after harvest owing to respiratory metabolism. As shown in Figure 8E, although a decline in TSS content was observed across all groups during storage, the reduction was most pronounced in the CK group (PVC film). In contrast, TSS retention in the composite film groups improved with increasing PPOE content before declining slightly, thereby effectively maintaining the flavor and quality of strawberries. This retention capacity of the CS-PPOE composite films can be attributed to their dense structure and low WVP, which effectively inhibit respiratory metabolism and minimize the consumption of soluble solids as respiratory substrates [42].

#### 3.3.12. Biodegradability of Composite Films

**Figure 9:**
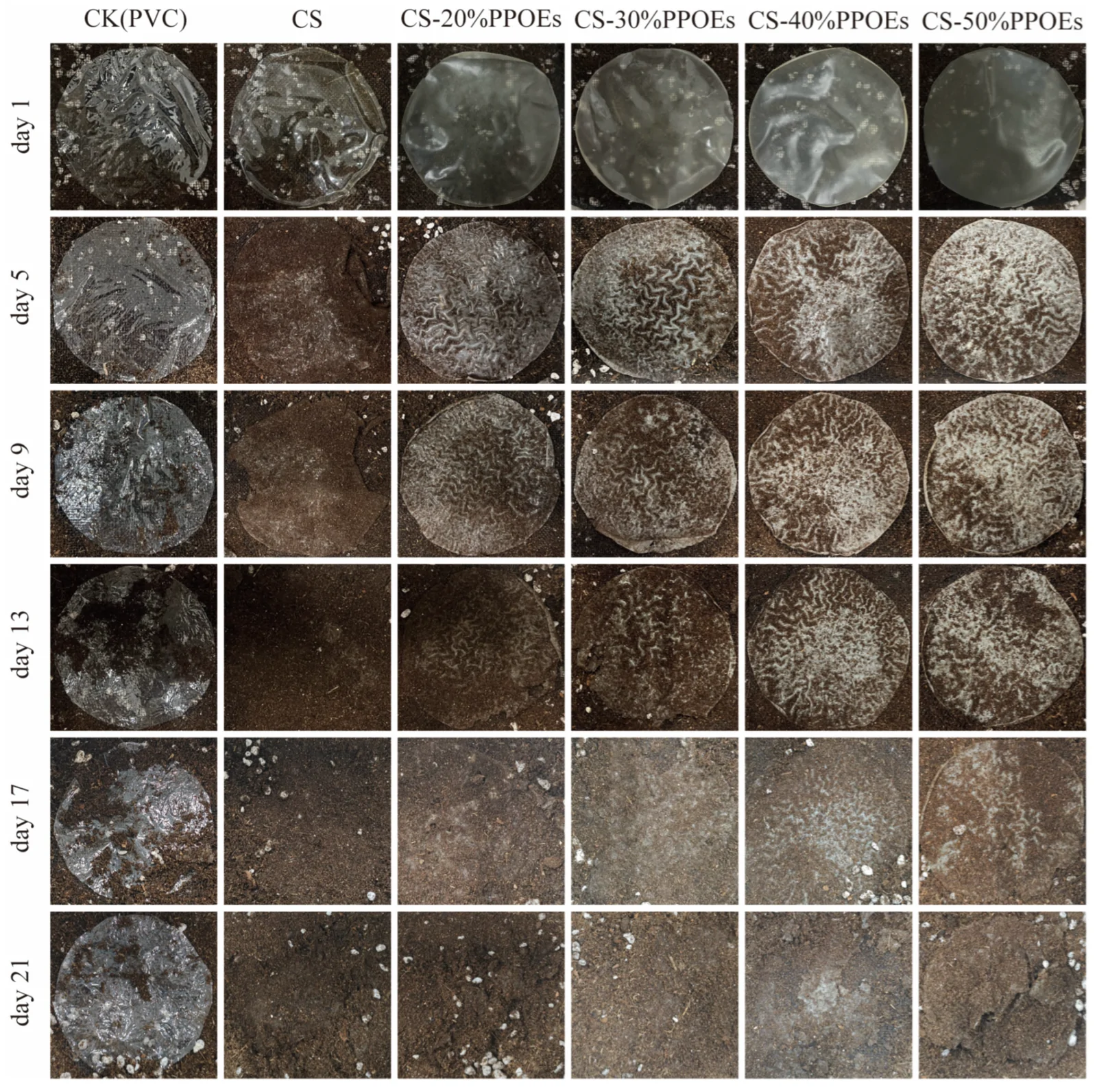
Degradability of composite films. Digital photographs taken on days 1, 5, 9, 13, 17 and 21.

All films, including the commercial PVC film (CK), were buried in moist soil for 21 days to evaluate biodegradability. As shown in Figure 9, the PVC film retained its structural integrity throughout the experiment, with no significant swelling, cracking, or fragmentation, except for slight moisture absorption, confirming the poor degradability of conventional petroleum-based plastics. This observation is consistent with previous reporting that other petroleum-based films remain structurally intact for ≥15 days under soil [52]. In contrast, the pure CS film degraded rapidly: intact on day 1, swollen and darkened on day 5, loosened on day 9, further collapsed on day 13, and almost completely degraded on day 21, leaving only minor debris. These findings confirm the excellent biodegradability of CS, as its polymer network is rapidly degraded by microbial enzyme action in soil, typically within a few weeks [49].

For composite films containing CS and different proportions of PPOEs, the degradation rate decreased progressively with increasing PPOE content. The CS-20% film degraded slightly more slowly than the pure CS film, remaining intact at 21 days. The CS-30% and CS-40% films exhibited a further slower degradation rate, with surface swelling and deformation observed on day 5; however, their basic outlines remained visible at 21 days. The CS-50% film degraded the slowest, largely preserving its structural integrity at 21 days [31]. This retardation in degradation is attributed to three factors. First, PPOE forms a denser hydrogen bond network with CS, enhancing structural stability against microbial attack. Second, the hydrophobic components of PPOEs reduce surface hydrophilicity, delaying hydrolysis [43,51]. Third, the broad-spectrum antimicrobial activity of PPOEs plays a dual role, inhibiting food surface microbes during application (for preservation) and temporarily suppressing soil-degrading microorganisms during initial burial, thereby slowing bioerosion [51]. All CS-PPOE composite films exhibited significant structural changes and partial degradation within 21 days, indicating good biodegradability, whereas the conventional PVC film remained intact. These results confirm that the prepared composite films, as disposable food packaging materials, can be degraded by environmental microorganisms after disposal, avoiding white pollution. Moreover, their degradation rate can be tuned by adjusting PPOE content to meet different food shelf-life requirements.

## 4. Conclusions

In this study, a stable Pickering emulsion (PPOEs) was successfully prepared using PPO and CIN as the oil phase (oil-to-water ratio, 1:50), with C-CNC and CS as composite stabilizers. The emulsion exhibited strong antibacterial activity. Incorporating PPOEs into the CS matrix significantly improved the UV resistance and the mechanical, thermal, and water-resistant properties of the composite films while maintaining good biodegradability. Strawberry preservation experiments indicated that the CS-PPOE film, particularly at 30% PPOEs, effectively delayed fruit decay, weight loss, softening, and TSS decline, thereby extending shelf life compared with the commercial PVC film (CK) and pure CS film.

Soil burial tests confirmed substantial degradation within 21 days. The present trials were limited to a single fruit species over a 5-day period, extending the evaluation to other perishable fruits, longer storage times, and real food matrices will further validate the practical applicability of this film. In addition, the specific contribution of C-CNC and CS to the interfacial stabilization of the Pickering emulsions and to the mechanical, barrier, and UV-shielding properties of the films and the chemical fate of cinnamaldehyde during and after film formation merits further investigation. A systematic comparison with the rigid plastic boxes and trays currently used for retail fruit packaging, in terms of mechanical performance, cost, and life-cycle assessment, will also be carried out in future work. Overall, the CS-PPOE composite film offers a promising sustainable alternative to petroleum-based plastics for the preservation of perishable fruits.

## Figures and Tables

**Table 1:** Inhibition zones of PPOEs against four common pathogenic bacteria at different concentrations.

Sample	H_2_O (mm)	20% PPOEs (mm)	40% PPOEs (mm)	60% PPOEs (mm)	80% PPOEs (mm)	100% PPOEs (mm)	1% CIN (mm)
*E. coli*	0 ^f^	12.71 ± 0.54 ^e^	18.04 ± 2.57 ^d^	22.14 ± 0.32 ^c^	27.78 ± 1.01 ^b^	32.98 ± 0.12 ^a^	21.80 ± 0.12 ^c^
*S. aureus*	0 ^f^	9.92 ± 0.21 ^e^	16.14 ± 0.71 ^d^	19.22 ± 0.36 ^c^	21.10 ± 1.10 ^b^	22.92 ± 1.12 ^a^	15.67 ± 0.15 ^d^
*L. monocytogenes*	0 ^f^	12.75 ± 0.50 ^e^	16.20 ± 0.79 ^d^	19.56 ± 0.28 ^c^	22.42 ± 0.27 ^b^	23.76 ± 0.29 ^a^	12.26 ± 0.62 ^e^
*V. parahaemolyticus*	0 ^e^	13.17 ± 0.43 ^d^	17.60 ± 0.56 ^c^	20.42 ± 0.37 ^b^	22.81 ± 1.13 ^a^	23.63 ± 0.39 ^a^	13.24 ± 0.20 ^d^

H_2_O, sterile distilled water (negative control); 1% CIN, 1% (*v*/*v*) cinnamaldehyde (positive control); 20–100% PPOEs, PPOEs diluted to the indicated volume fraction (*v*/*v*) with sterile distilled water. Different lowercase letters in the same row indicate significant differences (*p* < 0.05).

**Table 2:** Lightness (L*), redness (a*), yellowness (b*), and total color difference (ΔE) of composite films.

Sample	L*	a*	b*	ΔE
CS	49.47 ± 0.06 ^a^	0.10 ± 0.10 ^c^	14.43 ± 0.45 ^d^	51.32 ± 0.13 ^d^
CS-20%PPOEs	46.30 ± 0.10 ^b^	0.37 ± 0.06 ^b^	15.53 ± 0.95 ^cd^	54.68 ± 0.17 ^c^
CS-30%PPOEs	45.46 ± 0.23 ^c^	0.57 ± 0.06 ^a^	16.20 ± 0.61 ^bc^	55.66 ± 0.22 ^b^
CS-40%PPOEs	45.36 ± 0.23 ^c^	0.50 ± 0.10 ^ab^	17.03 ± 0.15 ^b^	56.00 ± 0.19 ^b^
CS-50%PPOEs	43.23 ± 0.32 ^d^	0.50 ± 0.10 ^ab^	18.50 ± 0.17 ^a^	58.48 ± 0.34 ^a^

Different lowercase letters in the same row indicate significant differences (*p* < 0.05).

## Data Availability

The original contributions presented in this study are included in the article. Further inquiries can be directed to the corresponding authors.
